# Genome Mining of the Marine Actinomycete *Streptomyces* sp. DUT11 and Discovery of Tunicamycins as Anti-complement Agents

**DOI:** 10.3389/fmicb.2018.01318

**Published:** 2018-06-20

**Authors:** Xiao-Na Xu, Liang-Yu Chen, Chao Chen, Ya-Jie Tang, Feng-Wu Bai, Chun Su, Xin-Qing Zhao

**Affiliations:** ^1^School of Life Sciences and Biotechnology, Dalian University of Technology, Dalian, China; ^2^College of Life Science, Dalian Minzu University, Dalian, China; ^3^Key Laboratory of Fermentation Engineering, Ministry of Education – Hubei Provincial Cooperative Innovation Center of Industrial Fermentation, Hubei Key Laboratory of Industrial Microbiology, Hubei University of Technology, Wuhan, China; ^4^State Key Laboratory of Microbial Metabolism, School of Life Sciences and Biotechnology, Shanghai Jiao Tong University, Shanghai, China; ^5^Key Laboratory of the Ministry of Education for Medicinal Resources and Natural Pharmaceutical Chemistry, College of Life Sciences, Shaanxi Normal University, Xi’an, China

**Keywords:** marine streptomycetes, genome mining, secondary metabolites, tunicamycin, anti-complement activity

## Abstract

Marine actinobacteria are potential producers of various secondary metabolites with diverse bioactivities. Among various bioactive compounds, anti-complement agents have received great interest for drug discovery to treat numerous diseases caused by inappropriate activation of the human complement system. However, marine streptomycetes producing anti-complement agents are still poorly explored. In this study, a marine-derived strain *Streptomyces* sp. DUT11 showing superior anti-complement activity was focused, and its genome sequence was analyzed. Gene clusters showing high similarities to that of tunicamycin and nonactin were identified, and their corresponding metabolites were also detected. Subsequently, tunicamycin I, V, and VII were isolated from *Streptomyces* sp. DUT11. Anti-complement assay showed that tunicamycin I, V, VII inhibited complement activation through the classic pathway, whereas no anti-complement activity of nonactin was detected. This is the first time that tunicamycins are reported to have such activity. In addition, genome analysis indicates that *Streptomyces* sp. DUT11 has the potential to produce novel lassopeptides and lantibiotics. These results suggest that marine *Streptomyces* are rich sources of anti-complement agents for drug discovery.

## Introduction

Marine environments cover more than 70% of the surface of the Earth, and are habitat of diverse microorganisms. Marine actinomycetes are rich sources for a myriad of bioactive natural products. Among various marine actinomycetes, marine streptomycetes have received the most attention due to their biosynthetic potential for producing novel bioactive compounds (Subramani and Aalbersberg, 2012; Agustina et al., 2016; Prietodavó et al., 2016).

Genome sequences of marine streptomycetes not only provide insights in mechanisms of marine environmental adaptation (Ian et al., 2014; Tian et al., 2016), but also benefit discovery of their biosynthetic potential (Zhao and Yang, 2011). Various useful metabolites were identified based on genome mining of *Streptomyces* (Xu et al., 2016; Ye et al., 2017; Yu et al., 2018). However, so far studies on genome mining of marine *Streptomyces* remain limited (Zhang et al., 2015; Chen et al., 2016; Jin et al., 2018).

Complement system is one of the important human immune defense systems, and it plays an important role in eliminating foreign microorganisms, clearance of damaged cells, adaptive immunity, inflammation, tissue regeneration, and tumor growth (Barnum, 2017). However, the improper activation of complement system may lead to a variety of diseases, such as rheumatoid arthritis and Alzheimer’s disease (Morgan and Harris, 2015). Chemical synthesis of anti-complementary agents has the limitations such as high cost, low selectivity, and unwanted side effects. Therefore, natural products with anti-complement activities have received increasing attention. Up to now, various anti-complementary compounds were isolated from algae and plants (Jin et al., 2015; Wen et al., 2017), whereas compounds with such activities have been poorly studied in microorganisms. The only known microbial-derived anti-complement compounds are complestatin and its analogs, which are produced by *Streptomyces lavendulae* SANK 60477 (Kaneko et al., 1989). Microbial production of anti-complement agents has the advantages to reduce production cost by rapid accumulation of metabolites and easy scale-up fermentation. In addition, novel compounds can also be obtained by genetic modifications of the microbial producers. Therefore, exploration of microbial strains with the potential to produce anti-complement compounds is of great interest, and the information of related genome sequences of the producer will promote rapid discovery of such active compounds.

During the screening of marine streptomycetes producing anti-complement compounds, we found that *Streptomyces* sp. DUT11 has superior anti-complement activities. In this study, the anti-complement compounds of *Streptomyces* sp. DUT11 were studied by genome mining of *Streptomyces* sp. DUT11, and its biosynthetic potential was also explored. Our current report indicates that marine *Streptomyces* strains are valuable sources of anti-complement compounds for drug discovery.

## Materials and Methods

### Strains and Culture Conditions

Strain *Streptomyces* sp. DUT11 was isolated from the marine sediment about 10 m below sea level in Xinghai Bay (39° 52′N, 121° 35′E), Dalian, China. This strain was deposited in the China General Microbiological, and the strain number was CGMCC 14581. The strain was maintained on ISP4 agar slants at 4°C and in 20% (v/v) glycerol stock at -80°C. For plate culture, strain DUT11 was grown at 28°C on Bennett’s agar. For seed culture, tryptic soy broth (TSB) medium was used.

To prepare samples for bioactivity assays, *Streptomyces* sp. DUT11 was inoculated into 20 mL TSB medium in 50 mL tubes to culture at 28°C for 2–3 days. Then, the seed culture was inoculated into the M33 fermentation media with an inoculum size of 2% (v/v) to culture at 28°C for 7 days. To prepare metabolites from agar culture for molecular networking analysis, 50 μL seed culture was spread over the 6 cm plate containing A1 agar medium to culture at 28°C for 7 days. The compositions of all the cultural media used in this study were provided in Supplementary Table S1.

### Genome Sequencing, Assembly, Annotation, and Mining

The genome of *Streptomyces* sp. DUT11 was sequenced through Pacbio RS technology (English et al., 2012) in State Key Laboratory of Microbial Metabolism, Shanghai Jiao Tong University, and the acquired data were assembled by Canu v1.5 (Berlin et al., 2015). The open reading frames (ORFs) prediction and genome annotation were acquired by RAST (Rapid Annotation using Subsystem Technology) (Brettin et al., 2015). The Clusters of Orthologous Groups (COGs) of gene functions and Kyoto Encyclopedia of Genes and Genomes (KEGG) pathway prediction was performed through WebMGA (Altschul et al., 1990; Ogata et al., 1999).

Identification of potential biosynthetic gene clusters (BGCs) for secondary metabolites were proposed by bioinformatics tool website antiSMASH (Weber et al., 2015). Further gene function analysis was through manual BLAST on NCBI and viewed on visual software Artemis Release 16.0 (Carver et al., 2012). The alignment of the genomes was performed by the website Double ACT v2^1^ and viewed through software Artemis Comparison Tool (ACT) (Carver et al., 2005). The genome sequence data have been deposited in the GenBank database and was assigned the accession number PRJNA351245 (CP025511).

### Sequence Analyses and Genome-Wide Comparative Analysis

16S rRNA PCR amplification was performed using the methods described previously (Lee et al., 2003; Li et al., 2007). The universal primers 27F (5′-AGAGTTTGATCCTGGCTCAG-3′) and 1429R (5′-AAGGAGGTGATCCAAGCCGCA-3′) were used to amplify the 16S rRNA sequence. The amplified 16S rRNA gene was sequenced by TaKaRa and uploaded to web-based EzTaxon-e program^2^ (Kim et al., 2012) for comparison. A 16S rRNA phylogenetic tree was created by software Geneious (Kearse et al., 2012) based on the EzTaxon-e database and partially on BLAST of some closely related strains.

The whole-genome based phylogenetic trees with peptide sequences or DNA sequences were generated with Composition Vector Tree v3 (CVtree v3) (Xu and Hao, 2009), and OrthoANI was used to generate OAT heat map (Ouk Kim et al., 2016). The genomes used for analysis were obtained from GenBank^3^. Nine closely related genomes were chosen for analysis. Due to the poor quality of the genome of *S. bacillaris* NBRC 13487, it was excluded in the genome alignments.

### Anti-complement Activity Test

Seven media, namely TSB, TSBY, TYDM, M3, M9, M33, and A1 medium (Supplementary Table S1) were chosen as fermentation media to test the production of anti-complement agents. The fermentation broth was centrifuged at 6,000 rpm for 15 min to obtain supernatant and mycelia. Subsequently, the supernatant was extracted by EtOAc and the mycelia was extracted by MeOH. Then the extracts were dried and re-dissolved into 1 mL 5% dimethyl sulfoxide (DMSO) solution to acquire crude extracts for anti-complement test.

A hemolytic assay was used to determine the inhibition of complement activation in the classical pathway (CP) (Morgan and Harris, 2015) with minor modifications. 1 × BBS (barbital buffer solution) buffer was used for solution buffer. The fresh sheep red blood cell (SRBC) was diluted to 2% for the test. Heparin was used as a positive control, which was dissolved in 1 × BBS. After preliminary test, 1/160 serum was chosen to be submaximal lysis in the absence of complement inhibitors. Sensitized erythrocytes (EAs) were prepared by incubating 2% SRBS with equal volumes of 1:1000 hemolysin. The samples were dissolved in 1 mL 5% DMSO, which were diluted 10 times in 5% DMSO to test anti-complement activities. For the anti-complement activity test, every 100 μL diluted sample and 100 μL serum solution (SS) were mixed and incubated at 37°C for 10 min, after which the mixture was cooled down on ice. Subsequently, 200 μL of EAs was added and the volume was filled to 600 μL with 1 × BBS. The mixtures were then incubated at 37°C for 30 min. Control groups were incubated under the same conditions, which include: (1) standard control: 200 μL EAs and 100 μL serum in 300 μL 1 × BBS; (2) 100% lysis: 200 μL EAs in water (400 μL); (3) sample blank: 100 μL dilution of each sample in 500 μL 1 × BBS; (4) sample test: 200 μL SS with 200 μL EAs in 200 μL 1 × BBS (Zhu et al., 2008). After incubation, the reacted mixtures were centrifuged at 5,000 rpm, 4°C for 10 min immediately. Optical density of the supernatants (200 μL) was measured at 405 nm with a spectrophotometer (Multiskan GO 1510, Thermo Fisher Scientific, Finland). The inhibition percentage of each sample was calculated by excluding the controls and blanks and then divided by a standard value, and the anti-complementary activity (%) is calculated from the formula (Wang et al., 2008):

$$\frac{{\text{A}}_{\text{control}}{\text{-A}}_{\text{sample}}}{{\text{A}}_{\text{control}}}\times \text{100}\%$$

### Extraction, Analysis, and Purification of the Compounds With Anti-complement Activity

For analysis the secondary metabolites in strain DUT11, the cultured plates were cut into small pieces and extracted with 1:1 MeOH/H_2_O or EtOAc. The samples were dried and re-dissolved into MeOH to analyze by UPLC-MTQ MS (Agilent1290- Bruker MicroTOF-Q II) using the gradient of 5–100% ACN/H_2_O containing 0.1% formic acid for 9 min, and 100% ACN with 0.1% formic acid for 1 min with a 0.5 mL/min flow rate (Agilent Infinity 1290 HPLC, Supelco Discover C18 250 mm × 4.6 mm, 5 μm column and Bruker MTQ MS system). The acquired LC-MS data was uploaded to Global Natural Products Social Molecular Networking (GNPS) website (Wang et al., 2016) to generate the molecular networking map and the map is viewed by Cytoscape v3.4. The open accession of the GNPS data in this study via MassIVE is ftp://massive.ucsd.edu/MSV000082328.

To isolate active components from fermentation broth and mycelia, *Streptomyces* sp. DUT11 was cultured in modified M33 medium at 30°C with 200 rpm agitation for 7 days for extraction and purification of the active compounds. After 7 days cultivation, the fermentation broth (45 L) was centrifuged at 6,000 rpm, after which the mycelia were extracted by MeOH and the supernatant was extracted by EtOAc. The resultant organic extracts were concentrated in vacuo until dried. The crude extracts of supernatant and mycelia were separated by C18 column with H_2_O/MeOH (v/v, 100:0, 70:30, 50:50, 30:70, 0:100), to afford five fractions for each samples. Fractions 4 and 4′ (70% MeOH) with high anti-complement activity were purified by HPLC on the C18 column (XDB-C18, 4.6 mm × 250 mm, 5 μm at a flow rate of 0.4 mL/min using a gradient solvent from 5 to 95% CH_3_CN with UV detector set at 260 nm, and compound A was isolated. The active fractions 5 and 5′ (100% MeOH) were purified using the same method to isolate compound B and compound C.

LC/MS (Agilent1290-MS6230 Trap system) was used for further detection of the compounds. LC/MS analysis was performed on a XDB-C18 Column (Agilent, 150 mm × 4.6 mm, 5 μm column) at a flow rate of 0.4 mL/min using a gradient solvent from 5 to 95% ACN over 50 min.

A Waters ACQUITY^TM^ series mass spectrometer equipped with an electrospray ionization source was used for UPLC-Q/TOF MS analysis of compounds. MS/MS detection mode was set as: mass range, from *m/z* 100 to 2,000 in positive mode; capillary, 3 kV; sample cone voltage, 35 V; desolvation gas temperature, 350°C; flow rate of desolvation gas, 600 L/h. The column temperature was maintained at 40°C. Mobile phase A (H_2_O, 0.1% formic acid) and B (ACN, 0.1% formic acid) were used; and the gradient program was as follows: 0–5 min 10% B, 30–31 min 40–10% B, 40 min 10% B; flow rate 0.35 mL/min; and injection volume was 1 μL.

## Results

### Comparative Analysis of the 16S rRNA and Genome of *Streptomyces* sp. DUT11

**FIGURE 1:**
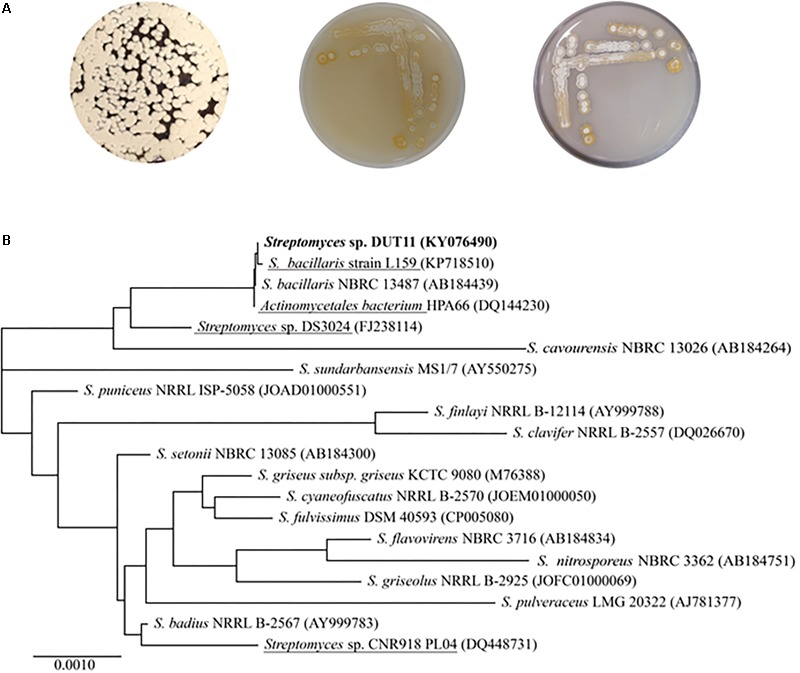
Morphological characteristics and phylogenetic analyses of *Streptomyces* sp. DUT11. **(A)** Phenotype of DUT11 cultured on A1 (left), M33 (middle) and ISP4 medium (right), respectively. **(B)** Neighbor-joining tree showing the phylogenetic relationships of DUT11 and related strains based on 16S rRNA sequences. The strains underlined were isolated from marine environment. The accession numbers of the 16S rRNA sequences were provided in the parentheses.

*Streptomyces* sp. DUT11 was selected due to its superior anti-complement activity during our primary assay of the marine strain library. This strain grows well on A1, M33, and ISP4 agar, generating white spores (**Figure 1A**). DUT11 tolerates up to 10% NaCl in TSB media, and achieved the highest biomass with 3% NaCl (data not shown). The 16S rRNA sequence of *Streptomyces* sp. DUT11 is the same with that of the type strain *S. bacillaris* NBRC 13487 (100% similarity), and is the most closest to that of *S. globisporus* NBRC 12867 (Waksman and Lechevalier, 1953) (99.65% similarity). However, so far the genome sequences of these two type strains are still not available (**Figure 1B**).

**FIGURE 2:**
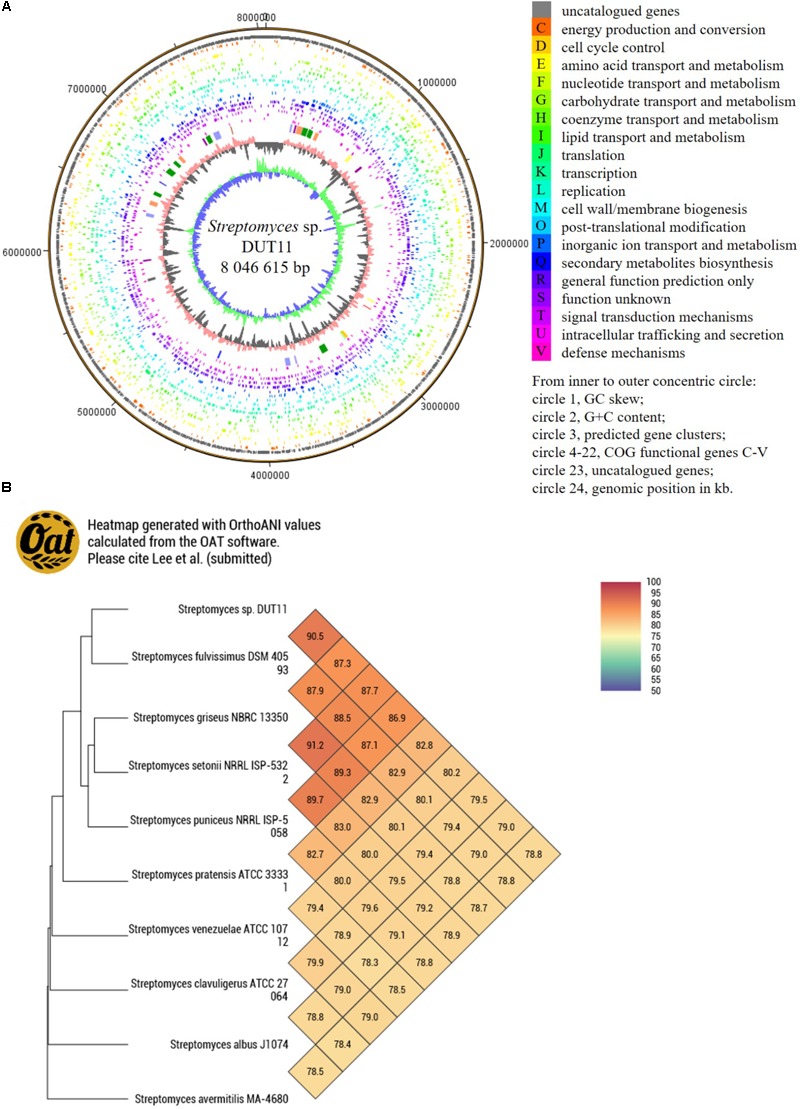
Circular map of *Streptomyces* sp. DUT11 genome and comparative genome analysis. **(A)** Circular map of the genome. **(B)** Heat map generated with OrthoANI values between strain DUT11 and nine closely related strains.

Due to the complicate procedures of anti-complement assay, we decided to use genome mining to facilitate the discovery of anti-complement agents from *Streptomyces* sp. DUT11. The genome of this strain was sequenced and analyzed. The genome size of *Streptomyces* sp. DUT11 is 8,027,164 bp with 71.83% GC content. There are 7,745 ORFs covering 6.76 Mb with 72.22% GC content. The coding percentage is 84.7% and the average length of ORFs is 872 bp. Totally 63 tRNA and 9 rRNA genes were also predicted. From the genome circle view, it was observed that the majority of the secondary metabolic BGCs were located near the ends of the genomes (**Figure 2A**). By blasting the COG database, two third of the annotated genes were classified, and there are 291 genes annotated to related with secondary metabolites (Supplementary Figure S1).

The genome sequence reported in this study thus provide basis to study related genomes. Among the closely related strains of DUT11, there are three marine actinomycetes, namely, *Streptomyces* sp. DS3024 (Hao et al., 2009), *S. bacillaris* strain L159 (Petříčková et al., 2015), and *Actinomycetales* bacterium HPA66 (Zhang et al., 2006). According the CVTree based on global protein or DNA sequences (Supplementary Figure S2), the most related strain with DUT11 is *S. fulvissimus* DSM 40593 (Myronovskyi et al., 2013), which shared a 16S rRNA similarity of 99.38% with DUT11. Furthermore, the OAT heat map (**Figure 2B**) also showed that *S. fulvissimus* DSM 40593 is the closest strain from genome scale, and the ANI (average nucleotide identity) score is 90.4% between the two strains. The second closest strain is *S. griseus* NBRC 13350 (87.9% similarity). The genomes of the rest strains are 78–83% similar to that of *Streptomyces* sp. DUT11.

### Analysis of the BGCs and Genome Mining for Discovery of Tunicamycins

There are 33 BGCs including at least seven non-ribosomal peptide synthases (NRPSs), five polyketide synthases (PKSs), six post-translationally modified peptides (PTMPs), and five terpene BGCs in the genome sequence. Furthermore, BGCs involve in the biosynthesis of ectoine, siderophore, bacteriocin, and butyrolactone which are common in most of the actinobacteria were also identified in DUT11 (**Table 1**).

**Table 1:** List of the BGCs in *Streptomyces* sp. DUT11.

No.	Type	Size (kb)	Most similar cluster (accession no., gene number)	Similarity (%)
1	Butyrolactone	1.9	Gamma-butyrolactone (AL645882, 2)	100
2	Terpene	22.1	–	
3	Other KS	62.2	Abyssomicin (MG243704)	10
4	NRPS	29.1	Griseobactin (FN545130, 17)	64
5	NRPS	20.6	Coelichelin (AL645882, 10)	72
6	T3PKS	2.7	Naringenin (CM000913, 2)	100
7	Lantipeptide	4.9	Venezuelin (HQ328852, 4)	100
8	Terpene	21.2	Steffimycin (AM156932)	19
9	Ectoine	4.6	Ectoine (AY524544, 4)	100
10	Lantipeptide	23.0	–	
11	Siderophore	6.5	Desferrioxamine B (AP009493, 5)	80
12	Thiopeptide	32.5	–	
13	Ectoine	10.4	–	
14	NRPS	67.5	Oxazolomycin (EF552687)	9
15	Ectoine-butyrolactone	15.4	Pristinamycin (FR681999)	17
16	Lantipeptide	7.6	Amf S (AP009493, 5)	100
17	Lassopeptide	5.0	SRO15-2005 (NZ_DS999644, 5)	100
18	Nucleoside	11.1	Tunicamycin (HQ172897, 14)	85
19	Terpene	21.1	–	
20	Siderophore-terpene	37.0	Kinamycin (AH012623)	8
21	T2 PKS	24.1	Medermycin (AB103463, 36)	66
22	NRPS	45.3	FD-594 (AB469194)	4
23	Bacteriocin	11.1	–	
24	NRPS	60.9	Asukamycin (GQ926890)	11
25	NRPS	43.8	Viomycin (AY263398)	9
26	T2 PKS	41.3	Nonactin (AF074603, 14)	92
27	Terpene	16.1	Hopene (AL645882, 13)	69
28	NRPS	55.0	Friulimicin (AJ488769)	6
29	T1 PKS-NRPS	21.7	SGR_PTMs (AP009493, 6)	100
30	Melanin	3.0	Melanin (AP009493, 2)	100
31	T3 PKS	1.7	Alkylresorcinol (AP009493, 3)	100
32	Terpene	8.1	Isorenieratene (AP009493, 7)	71
33	NRPS-thiopeptide	45.7	Daptomycin (AY787762)	10

*Similarity, the percentage of genes that are similar to genes in reference BGC.*

**FIGURE 3:**
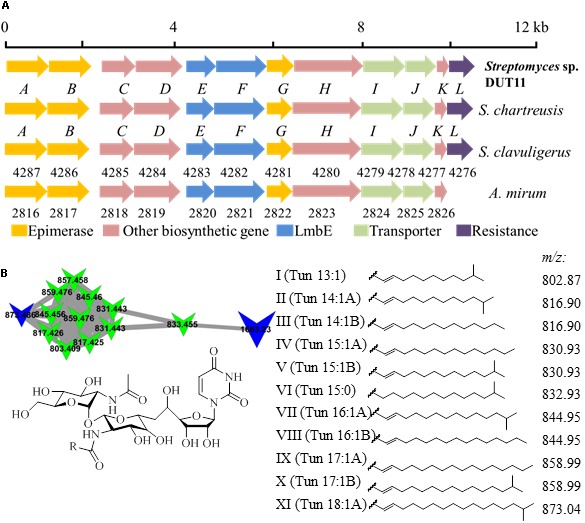
Organization of the tunicamycin BGC and identification of tunicamycins in *Streptomyces* sp. DUT11. **(A)** The tunicamycin BGC from strain DUT11. **(B)** Molecular networking map generated by GNPS and corresponding structures of the tunicamycin analogs.

Biosynthetic gene clusters potentially involved in biosynthesis of three known compounds, namely, tunicamycin, nonactin, and medermycin were focused. Among these BGCs, Cluster 18 was annotated as tunicamycin BGC by analyses with antiSMASH. Through further comparison with other tunicamycin BGCs in different strains, the cluster in DUT11 has the closest similarity to the gene cluster in *S. chartreuis* (Chen et al., 2010) (**Figure 3A**), and all the genes share around 94–98% identities with the corresponding genes in *S. chartreuis* (Supplementary Table S2). Strain DUT11 also lacks *tunM* and *tunN* which encodes methyltransferase and NUDIX hydrolase, respectively, but these genes are not essential for tunicamycin biosynthesis (Chen et al., 2010).

We further analyzed tunicamycin production in both agar culture and liquid fermentation. Firstly, we identified a cluster of tunicamycin analogs in GNPS map which has been annotated by GNPS (Wang et al., 2016). Depending on the reported structures and molecular weight range, at least eleven tunicamycin analogs with different lengths of alkane tails ranging from 13 to 18 carbons were identified from the A1 agar culture of DUT11 (**Figure 3B**) by comparing the MS spectrum with *m/z* shifts (Supplementary Figure S3).

### Discovery of Tunicamycins as Anti-complement Agents From *Streptomyces* sp. DUT11

Anti-complement activities of the extracts from both mycelia and fermentation broth of *Streptomyces* sp. DUT11 were detected. As shown in **Table 2**, when different culture media were compared, the highest anti-complement activities were observed using the M33 medium. In addition, similar anti-complement activities were observed for the extracts of supernatant and mycelia grown in M33 fermentation medium.

**Table 2:** Anti-complementary activities of *Streptomyces* sp. DUT11 in different fermentation media^∗^.

Sample type	Medium						
	TSB	TSBY	TYDM	M3	M9	M33	A1
S	44.0 ± 2.9	28.0 ± 5.0	12.0 ± 2.1	36.5 ± 3.6	30.2 ± 2.9	55.3 ± 1.5	39.9 ± 3.4
ES	45.3 ± 3.3	34.1 ± 4.7	13.1 ± 2.3	38.4 ± 3.8	31.1 ± 2.8	56.5 ± 2.0	44.2 ± 3.0
EM	47.2 ± 4.6	36.3 ± 4.0	14.1 ± 2.7	39.5 ± 3.0	32.9 ± 2.5	60.8 ± 2.2	46.2 ± 2.8

*^∗^S, supernatant; ES, extract of the supernatant; EM, extract of the mycelia. The values represent the percentages of inhibition (%); sample concentration was 0.06 mg/mL; the anti-complement activity of the positive control heparin at 0.06 mg/mL was 40.2%.*

**FIGURE 4:**
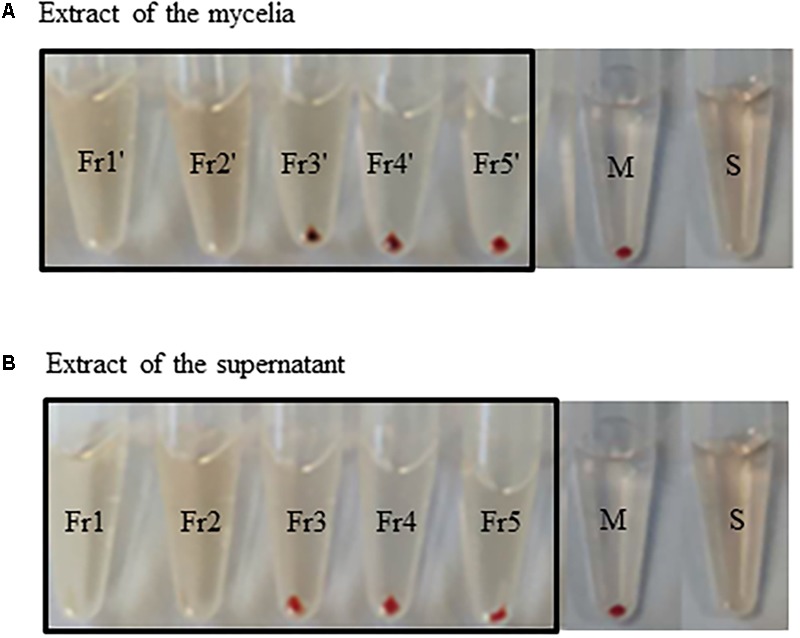
Anti-complement activity of the extracts of *Streptomyces* sp. DUT11. **(A)** The anti-complement activity of fractions from the extracts of mycelia obtained with different MeOH concentrations: Fr1′: H_2_O/MeOH (v/v, 100:0); Fr2′: H_2_O/MeOH (v/v, 70:30); Fr3′: H_2_O/MeOH (v/v, 50:50); Fr4′: H_2_O/MeOH (v/v, 30:70); Fr5′: H_2_O/MeOH (v/v, 0:100); S: full hemolysis; M: mechanical hemolysis. **(B)** The anti-complement activity of fractions from the extracts of supernatant. Fr1: H_2_O/MeOH (v/v, 100:0); Fr2: H_2_O/MeOH (v/v, 70:30); Fr3: H_2_O/MeOH (v/v, 50:50); Fr4: H_2_O/MeOH (v/v, 30:70); Fr5: H_2_O/MeOH (v/v, 0:100); S: full hemolysis control; M: mechanical hemolysis control.

We further isolated active fractions from the liquid culture using the M33 medium. The extracts of mycelia and supernatant were concentrated to yield 10 g and 8.5 g. The two organic extracts were separated by C18 column with H_2_O/MeOH (v/v, 100:0, 70:30, 50:50, 30:70, 0:100), to afford five fractions. The fractions 3, 4, and 5 from the crude extract of supernatant eluted using 50%, 70%, and 100% methanol showed good anti-complement activities, which were 58.9%, 77.1%, and 70.4%, respectively. Similarly, the fractions 3′, 4′, and 5′ eluted using 50%, 70%, and 100% methanol from the crude extracts of mycelia showed better anti-complement activity, which were 50.1%, 86.03%, and 58.6%, respectively. We deduced that the strain DUT11 can produce a variety of substances with anti-complement activity (**Figures 4A,B**).

**FIGURE 5:**
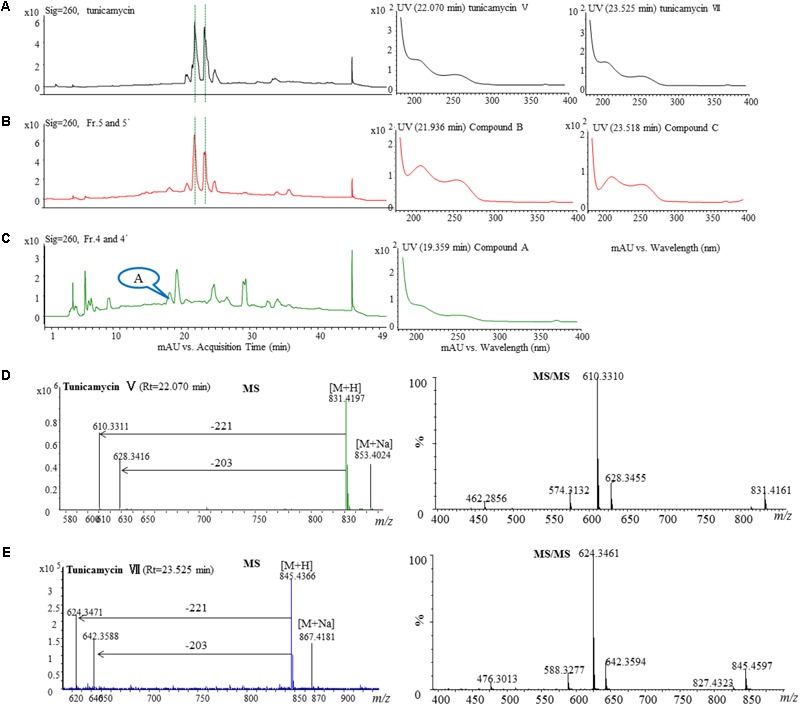
HPLC, MS, and MS/MS analysis of the tunicamycin standard and HPLC analysis of the active fractions. Each sample was purified by HPLC on the C18 column (XDB-C18, 4.6 mm × 250 mm, 5 μm at a flow rate of 0.4 mL/min using a gradient solvent from 5 to 95% CH_3_CN with UV detector set at 260 nm. **(A)** HPLC analysis of the tunicamycin standard under 260 nm and the UV-Vis spectra of the tunicamycin standard. **(B)** HPLC analysis of the active fractions 5 and 5′ under 260 nm and UV-Vis spectra of compounds B and C which were isolated from the fractions. The HPLC profiles of fraction 5 and 5’ are the same. **(C)** HPLC analysis of the active fractions 4 and 4′ under 260 nm and UV-Vis spectra of compound A which was isolated from the fractions. The HPLC profiles of fraction 5 and 5′ are the same. **(D,E)**, MS-MS analysis of the standard tunicamycin V and VII, respectively.

**FIGURE 6:**
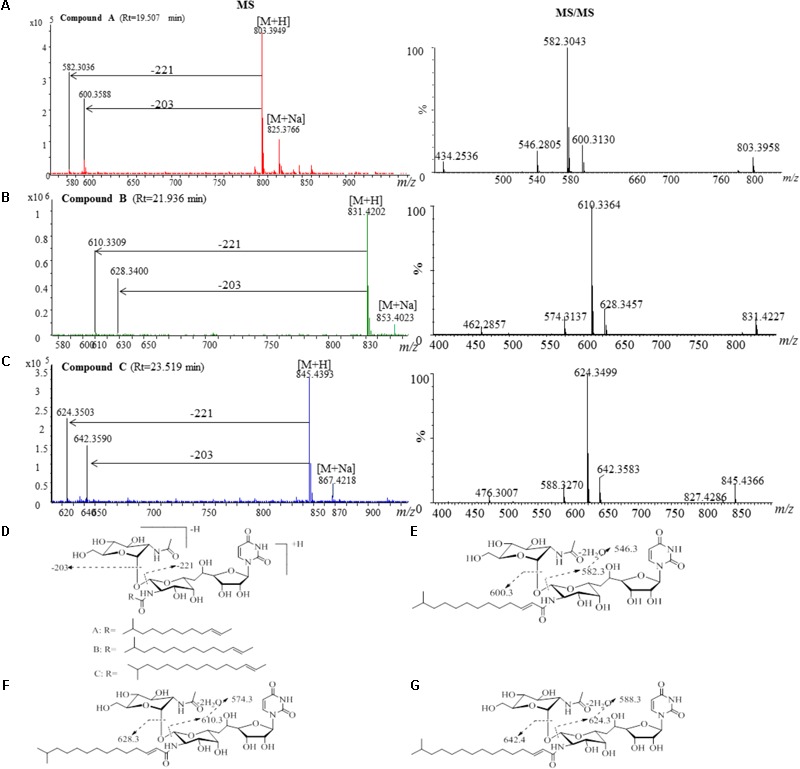
MS and MS/MS analysis of the purified compounds A, B and C. **(A)** The component of compound A generates a [M + H]^+^ ion at *m/z* 803.3926, which is fragmented into main ions of 600.3, 582.3, 546.3, etc. **(B)** The component of compound B generates a [M + H]^+^ ion at *m/z* 831.4141, which is fragmented into main ions of 628.3, 610.3, 574.3, etc. **(C)** The component of compound C generates a [M + H]^+^ ion at *m/z* 845.4366, which is fragmented into main ions of 642.4, 624.3, 588.3, etc. **(D)** Voltage gradient-induced ions at [M + H - 221]^+^ and [M + H - 203]^+^ indicate fragmentations across the compound A, B and C. MS/MS fragmentation pattern of the compound A **(E)**, compound B **(F)**, and compound C **(G)**.

The active fractions 4, 4′, 5, and 5′ were further analyzed by HPLC, and tunicamycins were detected. The profile of tunicamycin standard was presented in **Figure 5A**. Tunicamycin analogs compound A (retention time, Rt: 19.5 min), compound B (Rt: 21.9 min), and compound C (Rt: 23.5 min) were detected (**Figures 5B,C**) in the active fractions. In the tunicamycin standard we purchased, only two tunicamycin analogs, namely tunicamycin V and VII, which are corresponding to compounds B and C, were present (**Figure 5A**). MS/MS analysis of tunicamycin V and VII was shown in **Figures 5D,E**. We further purified the three tunicamycin analogs in the active fractions. Compound A gives [M + H]^+^/[M + Na]^+^ ions at *m/z* 803.3949/825.3766 (calcd. for C_36_H_59_N_4_O_16_: 803.3921, ppm: 3.49; C_36_H_58_N_4_O_16_Na: 825.3740, ppm: 3.15). Compound B gives [M + H]^+^/[M + Na]^+^ ions at *m/z* 831.4202/853.4023 (calcd. for C_38_H_63_N_4_O_16_: 831.4234, ppm: -3.84; C_38_H_62_N_4_O_16_Na: 853.4053, ppm: -3.52) and compound C gives [M + H]^+^/[M + Na]^+^ ions at *m/z* 845.4393/867.4218 (calcd. for C_39_H_65_N_4_O_16_: 845.4390, ppm: 0.35; C_39_H_64_N_4_O_16_Na: 867.4210, ppm: 0.92), respectively. The peaks generated by MS/MS analysis showed that the main fragment ions are at 600.3, 582.3, and 546.3; 628.3, 610.3, and 574.3; as well as 642.4, 624.3, and 588.3, respectively (**Figures 6A–C**). Fragmentation of molecular adduct ions give rise to [M + H-221]^+^ and [M + H-203]^+^ ions across the tunicamycin α β-1, 1′-glycosidic bonds to generate stable deglycosylated species, the relative masses of which are diagnostic of the attached *N*-acyl group (-R) (**Figure 6D**). Comparing with the structural spectroscopic data in the literature (Tsvetanova and Price, 2001; Chen et al., 2010) and the tunicamycin standard, we confirmed that compounds A, B, and C are tunicamycin I, V, and VII (**Figures 6E–G**), respectively. The other compounds in these active fractions are being investigated in our ongoing work.

**FIGURE 7:**
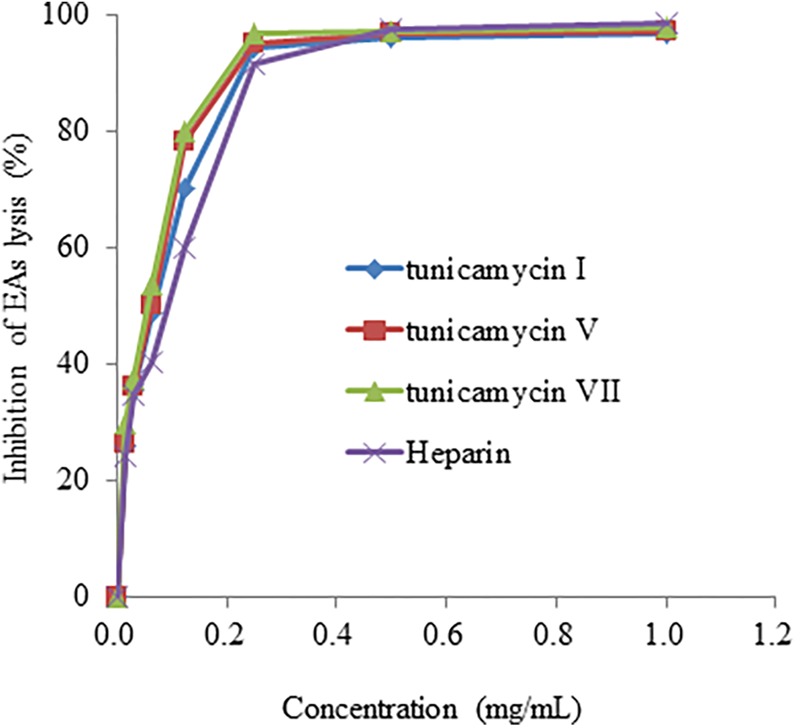
The anti-complement activity of tunicamycin I, V and VII. A hemolytic assay was used to determine the inhibition of complement activation in the classical pathway.

We further confirmed anti-complementary activities of the isolated tunicamycins produced by *Streptomyces* sp. DUT11. As shown in **Figure 7**, the concentrations that resulted in 50% hemolysis inhibition (CH_50_) of tunicamycin I, tunicamycin V, and tunicamycin VII were 0.071 ± 0.01 mg/mL (0.088 ± 0.012 mM), 0.060 ± 0.009 mg/mL (0.072 ± 0.011 mM), and 0.045 ± 0.009 mg/mL (0.053 ± 0.011 mM), respectively. The CH_50_ of the positive control heparin was 0.115 ± 0.05 mg/mL. The CH_50_ of tunicamycin standard V and VII were 0.059 ± 0.008 mg/mL (0.071 ± 0.009 mM), and 0.046 ± 0.009 mg/mL (0.054 ± 0.011 mM), respectively. This is the first time that tunicamycins are proved to have anti-complement activities.

### Other BGCs in *Streptomyces* sp. DUT11

Cluster 26 was originally identified as type II PKS gene cluster by antiSMASH. With further manual comparison, we found that the gene cluster is close to that of nonactin gene cluster from *S. griseus* ETH A7796 (Supplementary Figure S4A) (Walczak et al., 2000). Most of the genes in Cluster 26 shared 85–90% identities with that in the nonactin BGC (Supplementary Table S3). However, there is no counterpart of *non*I (AAD37458.1), which is a putative acetoacetate reductase in this gene cluster. In the primary search in the GNPS annotation, only monactin was found annotated in one molecular cluster. Subsequently, based on the information from GNPS and MS spectra comparison, seven cyclized nonactin analogs were found around the monactin node, including nonactin, dinactin, trinactin, and tetranactin (Smith et al., 2000) (Supplementary Figures S4B–D). Besides, several hydrolyzed liner nonactin analogs were also observed in the same molecular cluster. However, no anti-complement activity of nonactin was detected under our assay conditions in this study (data not shown).

Cluster 21 is another type II PKS BGC in DUT11, which has 66% similarity to that of the medermycin BGC from *Streptomyces* sp. K73 (Ichinose, 2003). The organization of Cluster 21 is also similar to the medermycin BGC. Nevertheless, the significant difference between the two BGC is that the two genes encoding phosphopantetheinyl transferase and putative carbohydrate kinase, respectively, are not present in Cluster 21. The other parts of the genes shares around 70–80% similarities between Cluster 21 and medermycin BGC (Supplementary Table S4). Due to the inability to get the medermycin standard sample, we did not test the anti-compliment activity of this compound.

**FIGURE 8:**
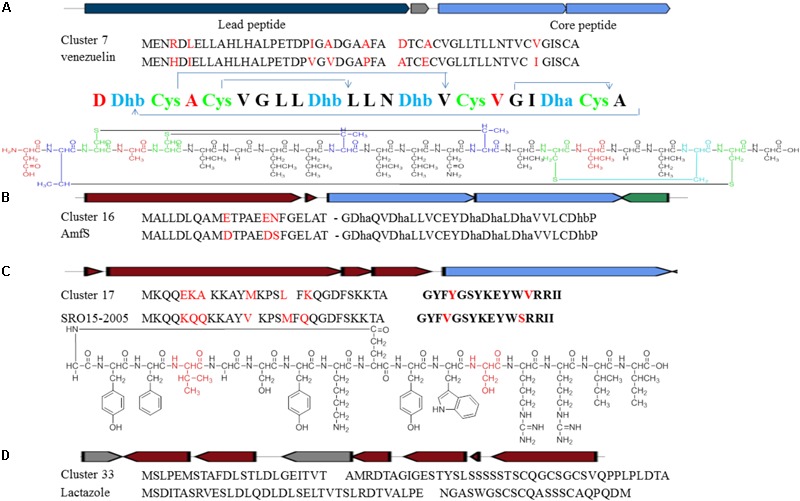
Identification of post-translationally modified peptide class BGCs in *Streptomyces* sp. DUT11. **(A)** Comparison of Cluster 7 with the venezuelin peptide gene cluster. **(B)** Comparison of Cluster 16 with the AmfS gene cluster. **(C)** Comparison of Cluster 17 with the lassopeptide SRO15-2005 gene cluster. **(D)** Comparison of Cluster 33 with the lactazole gene cluster.

Cluster 7 probably produces a lantibiotic peptide with four S-S cycles. The peptide sequence including leader peptide and core peptide is quite similar to the reported venezuelin peptide sequence (Kersten et al., 2011). However, five amino acid in the leader peptide and three in the core peptide are different (**Figure 8A**). The putative product of *ven*L in the reference BGC includes both protein kinase C (PK C) like superfamily domain and lanC-like superfamily domain. Nevertheless, DNA sequences encoding such two domains are divided into two separate genes in Cluster 7. The domains are highly conserved with around 72% similarities although the intergenic region is quite different.

Cluster 16 is a class III lantipeptide BGC and quite similar to AmfS BGC (**Figure 8B**), the product of which is a morphogen, a biological surfactant which positively regulates the formation of erect aerial mycelium (Ueda et al., 2002; Willey and Gaskell, 2011). The five genes involved in the biosynthesis share around 80% similarities with the reference ones. Compared to AmfS BGC, the core peptide encoded by related gene in this cluster is the same but the leader peptide contains three mutations.

Cluster 17 is a lassopeptide BGC and close to SRO15-2005 BGC (Maksimov et al., 2012) (**Figure 8C**). There are only two amino acid changes in the core peptide, but there are six amino acid differences in the leader peptide. The gene construction of Cluster 33 is partially close to lactazole BGC and might be a lassopeptide BGC hybrid with an additional NRPS and PKS (Hayashi et al., 2014) (**Figure 8D**). It is still not clear how the PKS and the NPRS affect the formation of the final products. The precursor peptides are quite different between the two BGCs, indicating that strain DUT11 has the potential to produce novel lassopeptides.

In addition, there are quite abundant BGCs of PTMPs in the DUT11 genome. Two of the tree lantibiotic BGCs and one lassopeptide found their close reference sequences which would help to identify the structures. Two more thiopeptide BGCs were identified as novel ones and the main backbone could be predicted.

Cluster 3 was supposed to be PKS I-NRPS hybrid BGC. There should be 7 PKS modules in this gene cluster and three A-domains of NRPS. This is the biggest PKS related BGC in the DUT11 genome. Through comparison and analysis, we found that there might be several key domains lost in the modules, and the backbone based on the function prediction was speculated. Another type III PKS BGC Cluster 6 might only include 2 genes and shared 71% similarities to naringenin BGC (Alvarez-Alvarez et al., 2015). The rest one was Cluster 29 and is a small type I PKS-NRPS hybrid gene cluster. This BGC only contains one PKS module including KS, AT, DH, KR, and ER domains and one A-domain responsible for ornithine. The BGC construction is quite close to the BGC class of polycyclic tetramate macrolactams including SGR PTMs BGC (Luo et al., 2013) and frontalamides BGC (Blodgett et al., 2010). Furthermore, eight proposed NRPS related BGCs were observed in the DUT11 genome, even though most of those only contained small NRPS genes. Cluster 4 and Cluster 5 were found to be quite close to BGC of griseobactin (Patzer and Braun, 2009) and coelichelin (Bentley et al., 2002), respectively. The predicted structures should be the same with reference compounds. Cluster 14 was found to be able to synthesize a four amino acid peptide backbone, namely, gly-ser-ser-ser. Cluster 24 was supposed to be a NRPS-PKS hybrid BGC which contains five A-domains coding leu-ser-dhb-cys-gly and one putative PKS sets. Further analysis of expression of these BGCs and their corresponding metabolites will provide functional insights of the *Streptomyces* sp. DUT11 genome.

## Discussion

Marine streptomycetes are rich sources of various secondary metabolites for drug discovery. In this study, we report the biosynthetic potential of the marine strain *Streptomyces* sp. DU11, which was revealed by analysis of its genome sequence. We found that genome sequences of many species having close phylogenetic relationship with stain DUT11 are still not available. Therefore, the available genome information reported in this study will assist the studies of these closely related species.

Complement system plays important roles in defense of invading microorganisms, clearance of damaged cells, adaptive immunity, and tissue regeneration (Hourcade et al., 2016). However, inappropriate and excessive activation of complement system will cause tissue damage diseases, such as systemic lupus erythematosus (SLE), rheumatoid arthritis (RA), systemic inflammatory response syndrome (SIRS), and acute respiratory distress syndrome (ARDS) (Qu et al., 2009). Anti-complement compounds are potential drugs to cure disorder of complement system, but so far such agents are mainly from plants rather than microbial source (Kaneko et al., 1989). Tunicamycins are a family of nucleoside antibiotic consisting of uracil, *N*-acetylglucosamine, a unique 11-carbon 2-aminodiadose sugar (tunicamine), and an *N*-acyl chain with variable lengths (Tsvetanova and Price, 2001; Price and Tsvetanova, 2007). Tunicamycins target bacterial cell wall biosynthesis by inhibiting early stage of peptidoglycan biosynthesis (Winn et al., 2010). They are inhibitors of eukaryotic protein *N*-glycosylation to induce endoplasmic reticulum stress and consequently become drugs to study apoptotic cell death related diseases (Cóppola-Segovia et al., 2016; Guo et al., 2017). The antagonistic activity of tunicamycin against *Bacillus subtilis* was known (Chen et al., 2010). In the previous study, tunicamycin was proved to be able to block the glycosylation on the synthesis of pro-C4, C2 and factor B and inhibited the secretion of these proteins, which led to decrease of the activity of the complement system, and the study was performed using tissue culture of guinea-pig peritoneal macrophages (Matthews et al., 1982), where the authors incubated for 6 h in the experiment. However, in our current study, we added tunicamycins directly to combine complement proteins *in vitro* to inhibit the complement complex activity, and our assay is more rapid (less than 1 h). We found that tunicamycin I, V and VII have anti-complement activity comparable to, if not stronger, than other natural anti-complementary agents (**Table 3**). Although complestatins show better anti-complement activity, these compounds have very poor solubility. Therefore, searching for new microbial-derived anti-complement agents are of great interest. Our results suggest that other compounds with known structures may also be explored to their anti-complement activities.

**Table 3:** The anti-complementary activities of other compounds on the classical pathway (CH_50_)^∗^.

Compound	Anti-complementary activity (CH_50_ mM)	Source	Reference
Stigmasta-4-ene-3β, 6β-diol	0.060 ± 0.020	*Viola kunawarensis*	Wang et al., 2017
Saringosterone	0.080 ± 0.030		
Aurantiamide acetate	0.020 ± 0.010		
Solalyratin B	0.050 ± 0.020		
Machicendonal	0.040 ± 0.009	*Helicteres angustifolia*	Yin et al., 2016
(*7S,8R*)-dihydrodehydrodiconiferyl Alcohol	0.009 ± 0.002		
Fifteen cycloartane triterpenes	0.120 to 0.467	*Beesia calthaefolia*	Mu et al., 2016
Eleven phenolic compounds	0.113 to 1.210	*Viola tianshanica*	Qin et al., 2015
Isomangostanin	0.032 ± 0.009	*Garcinia mangostana*	Quan et al., 2010
Garcinone E	0.012 ± 0.012		
(*3S*)-falcarinol	0.087	*Dendropanax morbifera*	Chung et al., 2011
(*3S, 8S*)-falcarindiol	0.015		
(*3S*)-diynene	0.040		
Complestatin	0.0003	*Streptomyces lavendulae* SANK 60477	Kaneko et al., 1989
Tunicamycin I	0.088 ± 0.012	*Streptomyces* sp. DUT11	This study
Tunicamycin V	0.072 ± 0.011		
Tunicamycin VII	0.053 ± 0.011		

*^∗^CH_*50*_ means the concentrations that resulted in 50% hemolysis inhibition.*

Nonactin and related analogs are a central class of macrocyclic ionophores consisting of 32-membered ring (a cyclodotricontane) built with 24 carbon and 8 oxygen atoms (Martinez-Haya et al., 2017). These compounds show antibiotic, antitumor and anti-virus activities and are also widely used for the preparation of ion-selective electrodes and sensors (Zhan and Zheng, 2016). Although production of nonactin has been attempted through chemical synthesis and optimization of biosynthesis, the yields are not satisfied so far (Zhan and Zheng, 2016). Therefore, manipulation of strain DUT11 may provide alternative source of nonactin and its analogs.

Medermycin is featured with a fused three-ring structure composed of a benzene ring, a quinone and a stereospecific pyran ring, and possesses significant antitumor activities against many types of cancer cells as well as antibacterial activity (He et al., 2015; Lü et al., 2015). Our studies indicate that DUT11 is a promising producer of medermycin for drug discovery.

Genome mining and metabolite analysis indicates that strain DUT11 has great potential in secondary metabolite production. Besides the production of tunicamycin, nonactin and medermycin analogs with known structures, many other BGCs in strain DUT11 may produce novel metabolites. For example, there are quite abundant PTMP BGCs in the genome sequence of DUT11. PTMPs are ribosomal peptides with various activities including antimicrobial, antitumor and antivirus activities (Arnison et al., 2012). We have found that the extracts of mycelia and supernatant of strain DUT11 have good antifungal and antibacterial activities (Supplementary Figure S5), and the novel PTMPs would be further explored to produce such bioactive compounds. Furthermore, up to four NRPS BGCs, three PKS BGCs and four terpene BGCs are quite different from the known BGCs in the literature, and novel metabolites may be produced by further genome mining.

From the phylogenetic tree based on 16S rRNA sequences, strain DUT11 is close to *S. bacillaris*, and up to now, the only active compound was reported in *S. bacillaris* strain L159 (Hu and Macmillan, 2012), which revealed a novel peptide with autophagy inhibitory activity. There is no other report about the genome analysis and potential analysis of secondary metabolites produced by the closely related strains of DUT11. Therefore, exploration of the novel metabolites in DUT11 will provide more insights on the biosynthetic potential of marine microorganisms.

*Streptomyces* sp. DUT11 tolerates up to 10% NaCl and grows well in 3% NaCl in the liquid TSB medium (Supplementary Figure S6). Its fast growth and ability of producing various secondary metabolites make it suitable to be further explored to produce useful metabolites using sea water. Our studies here indicate that marine streptomycetes are valuable sources for developing anti-complement agents and other novel metabolites for biotechnological applications.

## Author Contributions

CS, F-WB, and X-QZ conceived the project. X-NX and L-YC performed the study. X-NX, L-YC, and CC analyzed the data and drafted the manuscript. X-QZ, CS, and Y-JT critically revised the manuscript. All authors read and approved the final manuscript.

## Conflict of Interest Statement

The authors declare that the research was conducted in the absence of any commercial or financial relationships that could be construed as a potential conflict of interest.

## Footnotes

1 http://www.hpa-bioinfotools.org.uk/pise/double_actv2.html

2 http://eztaxon-e.ezbiocloud.net/

3 http://www.ncbi.nlm.nih.gov/genome/
