# Supplementary material for: Genome Mining of the Marine Actinomycete Streptomyces sp. DUT11 and Discovery of Tunicamycins as Anti-complement Agents
Source: Front Microbiol. 2018 Jun 20;9:1318. doi: 10.3389/fmicb.2018.01318 (PMC6019454; doi:10.3389/fmicb.2018.01318)
Supplement: Supplementary file 1 [file Data_Sheet_1.docx]

**Supplementary data for**

**Genome mining of the marine actinomycete *Streptomyces* sp. DUT11 and discovery of tunicamycins as anti-complement agents**

Xiaona Xu^1#^, Liangyu Chen^1#^, Yajie Tang^4^, Fengwu Bai^2^, Chao Chen^5^, Chun Su^3*^, Xinqing Zhao^2*^

*^1^School of Life Science and Biotechnology, Dalian University of Technology, Dalian 116024, Liaoning, China.*

*^2^State Key Laboratory of Microbial Metabolism, School of Life Sciences and Biotechnology, Shanghai Jiao Tong University, Shanghai 200240, China.*

*^3^National Engineering Laboratory for Resource Developing of Endangered Chinese Crude Drugs in Northwest China, College of Life Sciences, Shaanxi Normal University, Xi’an 710119, China.*

*^4^Key Laboratory of Fermentation Engineering (Ministry of Education), Hubei Provincial Cooperative Innovation Center of Industrial Fermentation, Hubei Key Laboratory of Industrial Microbiology, Hubei University of Technology, Wuhan 430068 China.*

*^5^College of Life Science, Dalian Nationalities University, Dalian, 116600, Liaoning, China.*

**Supplementary figure legends**

**Fig. S1** **Numbers of different functional genes characterized by COG database.**

**Fig. S2 Composition Vector Trees (CV Trees) showing the phylogenetic relationships of *Streptomyces* sp. DUT11 and related strains based on whole-genome sequences.** (A) and (B), the CV Tree based on protein sequence and DNA sequence, respectively. The accession numbers were provided in the parentheses.

**Fig. S3 MS/MS spectrums of tunicamycin I-XI and its related analogues.** The samples were dried and re-dissolved in MeOH to analyze by UPLC-MTQ MS (Agilent1290- Bruker MicroTOF-Q II).

**Fig. S4** **BGC and molecular networking of nonactin analogues in** ***Streptomyces* sp.**  **DUT11.** (A) Identified gene cluster and reported nonactin gene cluster. (B) Molecular networking map of nonactin analogues. (C) Corresponding structures of nonactins. (D) MS/MS spectra of nonactin and its related analogues. The samples were dried and re-dissolved in MeOH to analyze by UPLC-MTQ MS (Agilent1290-Bruker MicroTOF-Q II).

**Fig. S5 Antimicrobial activities of *Streptomyces* sp. DUT11.** *Saccharomyces cerevisiae* BY4741, *Aspergillus niger* CBS 513.88 and *Candida albicans* CGMCC 2.538 were used as test strains to investigate anti-fungal activities of *Streptomyces* sp. DUT11. *Staphyloccocus aureus* ATCC 29213, *Escherichia coli* ATCC35218, *Bacillus subtilis* ATCC 6633 and *Pseudomonas aeruginosa* ATCC27853 were chosen to investigate the anti-bacterial activities of *Streptomyces* sp. DUT11. All the fungal strains were cultivated in YPD liquid medium and all the bacterial strains were cultured in LB medium. The crude extracts diluted in MeOH were used for the test, and 100 μL MeOH was used as a control. The diameter of the inhibition zone was measured after 48 hours incubation at 30 °C (fungi) or after 24 hours incubation at 37 °C (bacteria).

**Fig. S6 Growth of *Streptomyces* sp. DUT11 grown in different concentrations of NaCl.** The strain was grown in TSB liquid media with 0%, 3%, 5%, 8%, 10% or 15% NaCl and cultured at 28 °C for 5 days. Subsequently, 1 mL culture broth was centrifuged at 6000 rpm for 15 min to collect the mycelia, which were dried at 45 °C for 12 h and the dry weight of mycelia was weighed.


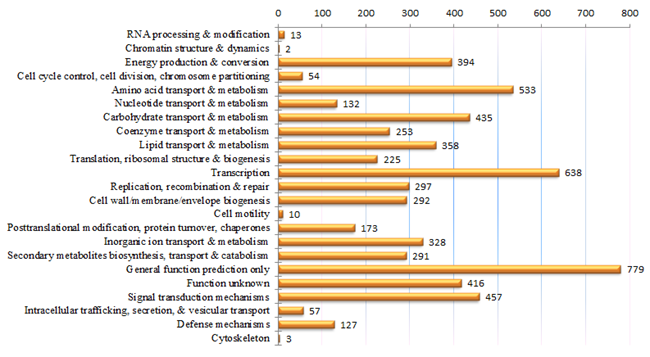


**Fig. S1**

**A**


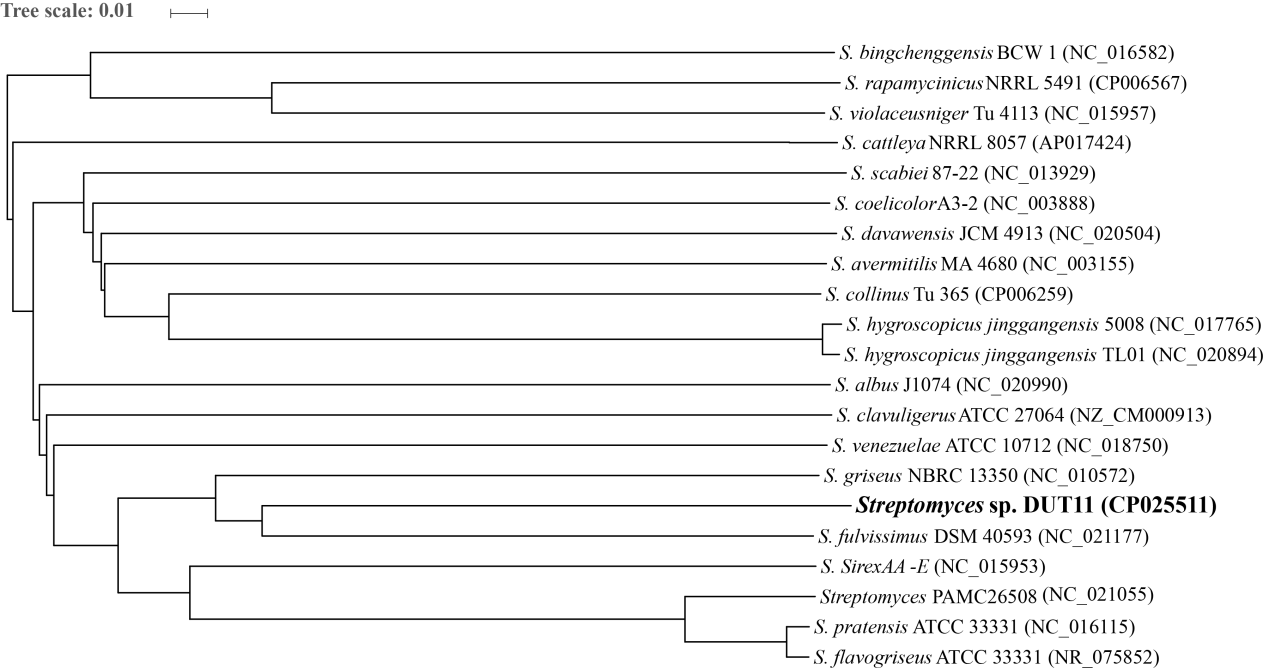


**B**


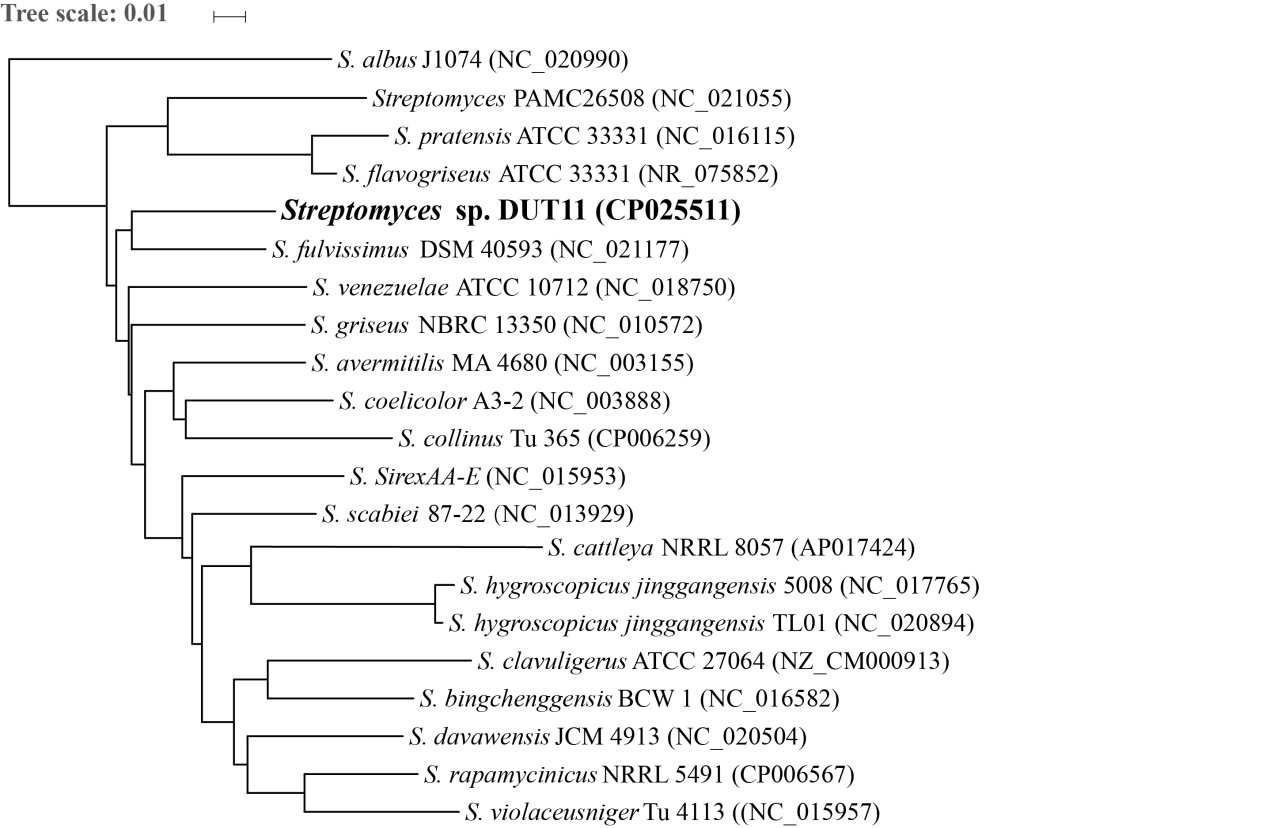


**Fig. S2**


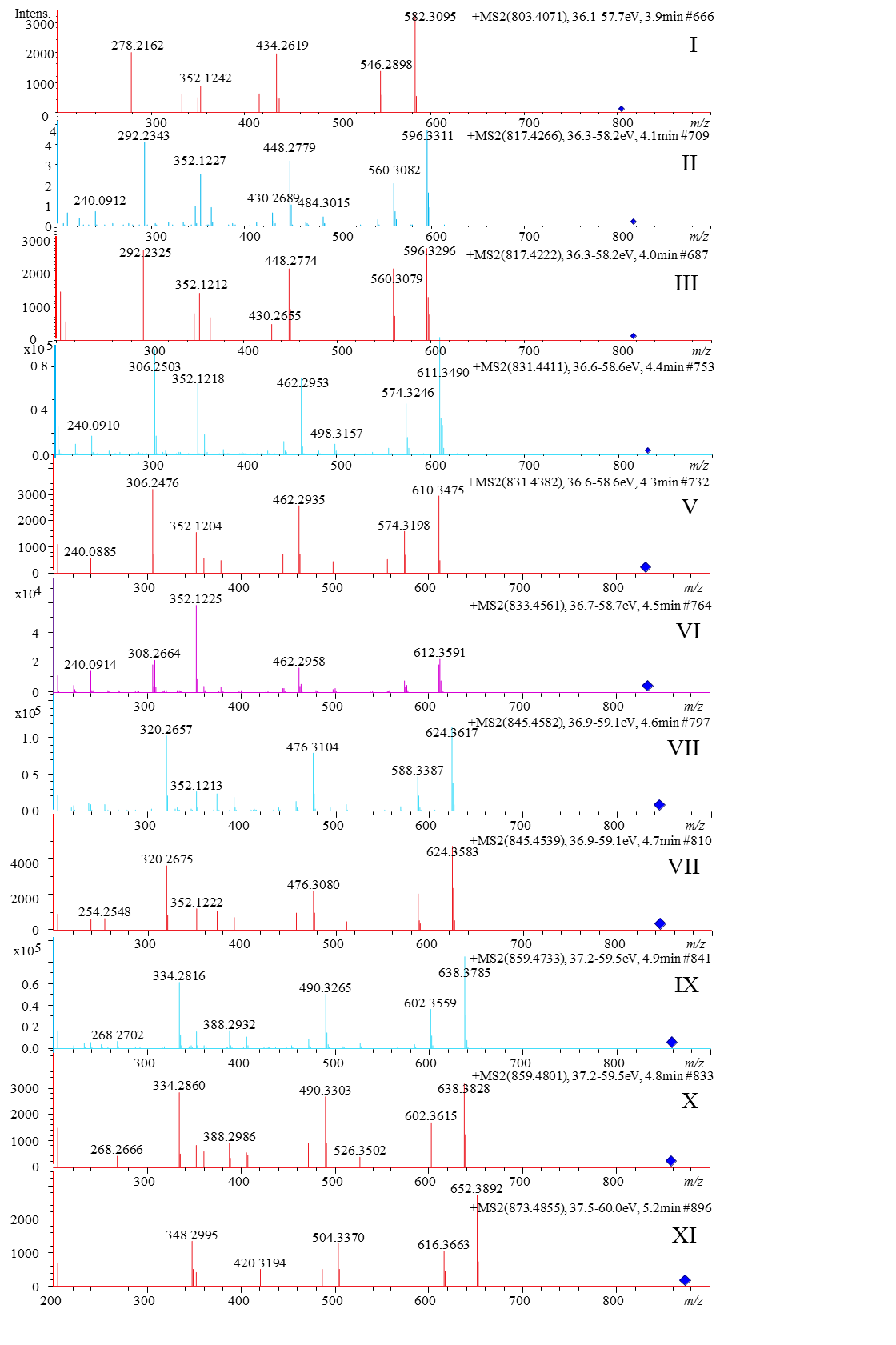


**Fig. S3**

**
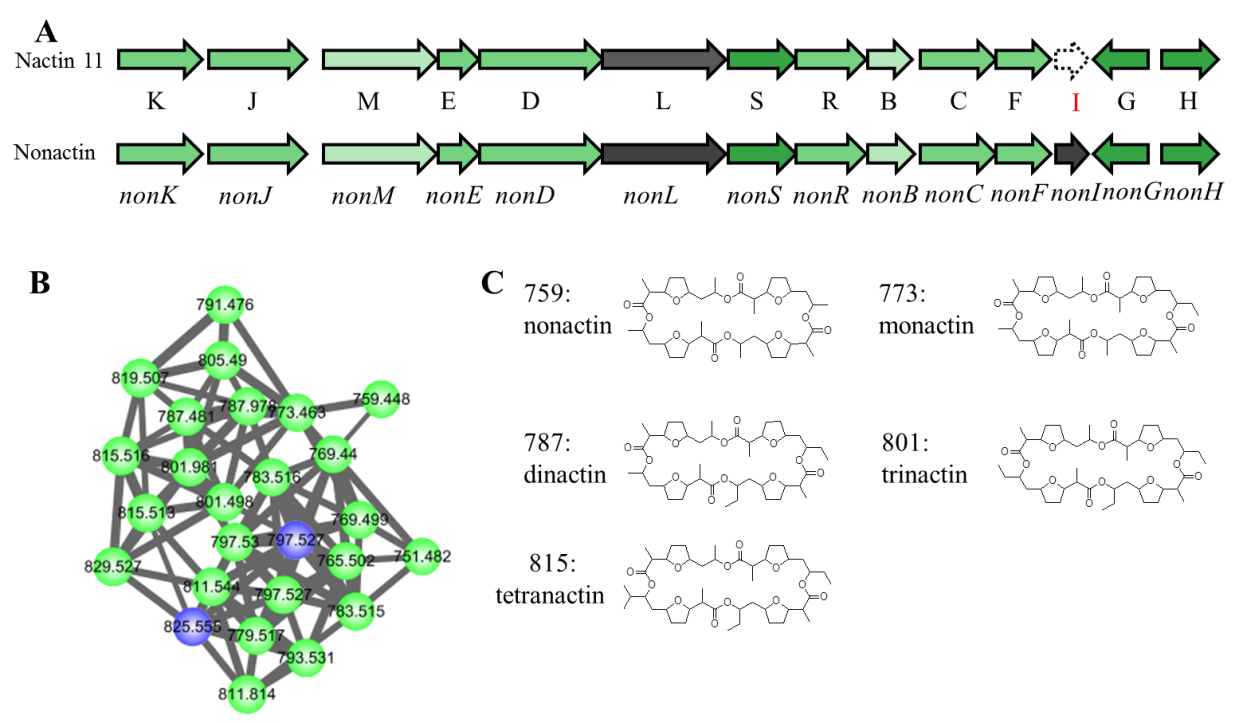
**

**D**

**
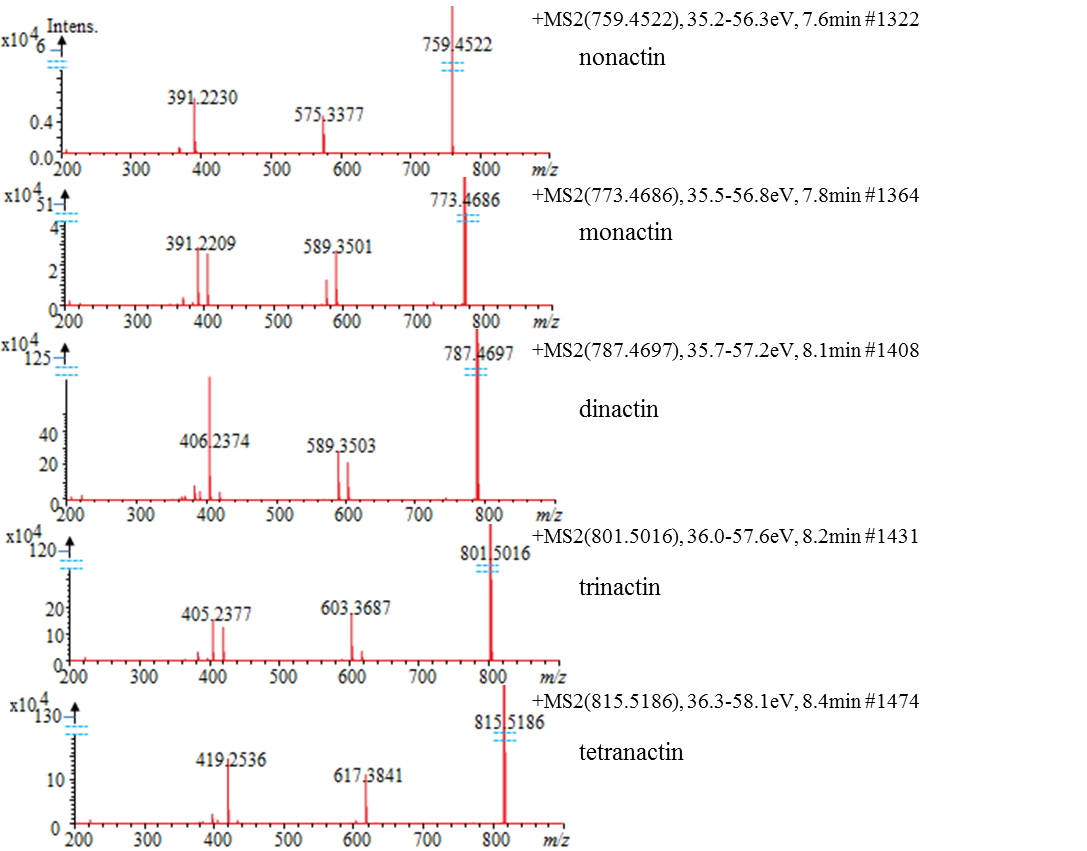
**

**Fig. S4**


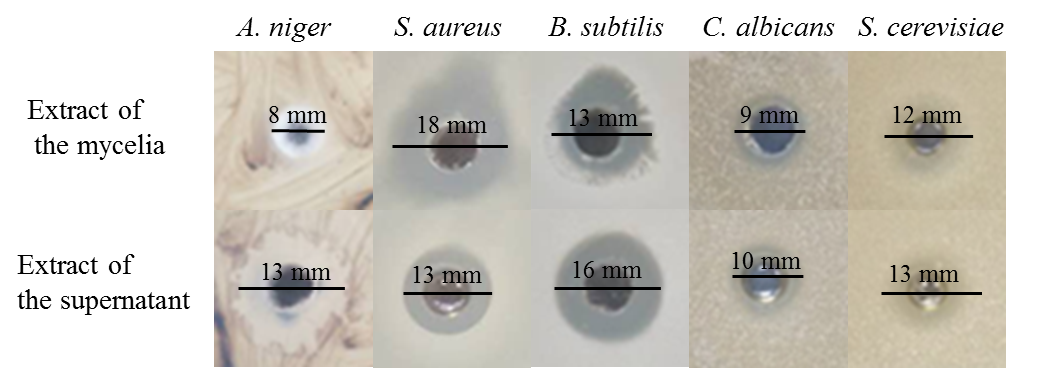


**Fig. S5**

The dry weight of mycelium (g/L)

**Fig. S6**

**Supplementary tables**

### Table S1. Culture media used in this study.

| Medium name | Medium composition (1 L) |
| --- | --- |
| TSB | tryptone 17 g, phytone 3 g, glucose 2.5 g, K_2_HPO_4_ 2.5 g, NaCl 10 g, pH 7.0 |
| A1 | yeast extract 4 g, soluble starch 10 g, peptone 2 g, aquarium salt 28 g, agar 15 g, pH natural |
| TSBY | TSB, yeast extract 5 g, sucrose 103 g, pH natural |
| TYDM | casein peptone 2 g, yeast extract 2 g, sucrose 10 g, MnCl_2_·6H_2_O 0.3 g, pH natural |
| M3 | soybean powder 25 g, soluble starch 20 g, (NH_4_)_2_SO_4_ 2 g, NaCl 2 g, K_2_HPO_4_ 0.5 g, CaCO_3_ 5 g, pH 7.2 |
| M9 | sucrose 20 g, soybean powder 10 g, corn flour 10 g, KCl 8 g, pH 6.5 |
| M33 | soybean powder 10 g, soluble starch 20 g, yeast extract 2.5 g, CaCO_3_ 3 g pH 7.0 |
| ISP4 | Soluble starch 10.0, K_2_HPO_4_ 1.0, MgSO_4_·7H_2_O 1.0, NaCl 1.0, (NH_4_)_2_SO_4_ 2.0, CaCO_3_ 2.0, trace salt solution 1.0 ml, agar 15 g, pH 7.0 |
| YPD | peptone 20 g, yeast extract 10 g, glucose 20 g, agar 15 g, pH 7.0 |
| LB | tryptone 10 g, yeast extract 5 g, NaCl 10 g, agar 15 g, pH 7.0 |

### Table S2. Deduced functions of the open reading frames in the tunicamycin BGC in *Streptomyces* sp. DUT11.

| Gene name | Related gene | Identities  (%) | Proposed function | Sequence ID |
| --- | --- | --- | --- | --- |
| Gene_6732 | *tun*A | 90 | UDP-GlcNAc epimerase | WP_010045816.1 |
| Gene_6731 | *tun*B | 97 | epimerase | WP_010045814.1 |
| Gene_6730 | *tun*C | 99 | radical SAM protein | WP_010045812.1 |
| Gene_6729 | *tun*D | 97 | glycosyl transferase family 1 | WP_010045809.1 |
| Gene_6728 | *tun*E | 96 | LmbE family protein | WP_010045808.1 |
| Gene_6727 | *tun*F | 97 | LmbE family protein | WP_010045808.1 |
| Gene_6726 | *tun*G | 96 | UDP-glucose 4-epimerase GalE | WP_010045806.1 |
| Gene_6725 | *tun*H | 94 | nucleotide pyrophosphatase | WP_029181580.1 |
| Gene_6724 | *tun*I | 96 | ATP-binding protein | WP_010045795.1 |
| Gene_6723 | *tun*J | 99 | ABC transporter | WP_010045793.1 |
| Gene_6722 | *tun*K | 99 | acyl carrier protein | WP_010045791.1 |
| Gene_6721 | *tun*L | 94 | phosphoesterase PA-phosphatase-like protein | WP_010045787.1 |

### Table S3. Deduced functions of the open reading frames in the nonactin BGC in *Streptomyces* sp. DUT11^*^.

| Gene name | Identity  (%) | Coverage | Subject gene | Proposed function |
| --- | --- | --- | --- | --- |
| Gene_8190 | 97 | 91.5 | AAD37460.1 | putative flavoprotein reductase |
| Gene_8191 | 85 | 100.0 | AAD37459.1 | putative transcriptional repressor |
| Gene_8193 | 87 | 100.0 | AAD37457.1 | NonF |
| Gene_8194 | 88 | 93.8 | AAD37456.1 | NonC |
| Gene_8195 | 94 | 100.0 | AAD37455.1 | NonB |
| Gene_8196 | 87 | 92.1 | AAD37454.1 | tetranactin resistance protein |
| Gene_8197 | 91 | 99.0 | AAC26135.1 | nonactate synthase |
| Gene_8198 | 92 | 98.8 | AAC26134.1 | putative nonactic acid:CoASH ligase |
| Gene_8199 | 89 | 100.0 | AAC26133.1 | putative diester hydrolase |
| Gene_8200 | 90 | 100.0 | AAD37453.1 | putative ketoacyl reductase |
| Gene_8201 | 87 | 82.3 | AAD37452.1 | putative ketoacyl reductase |
| Gene_8202 | 93 | 100.0 | AAD37451.1 | NonJ |
| Gene_8203 | 94 | 100.0 | AAD37450.1 | NonK |

### Table S4. Deduced functions of the open reading frames in the medermycin gene cluster in *Streptomyces* sp. DUT11.

| Gene name | Identity  (%) | Coverage | Subject gene | Proposed function |
| --- | --- | --- | --- | --- |
| Gene_6213 | 86 | 100.0 | BAC79022.1 | Tet-R family transcriptional regulator |
| Gene_6214 | 57 | 100.0 | BAC79023.1 | unknown |
| Gene_6215 | 86 | 100.0 | BAC79024.1 | acyl carrier protein (ACP) |
| Gene_6216 | 91 | 100.0 | BAC79026.1 | decarboxylase |
| Gene_6217 | 89 | 100.0 | BAC79027.1 | first ring aromatase (ARO) |
| Gene_6218 | 92 | 100.0 | BAC79028.1 | NDP-deoxyhexose 3-aminotransferase |
| Gene_6219 | 87 | 100.0 | BAC79029.1 | NDP-1-glucose synthase |
| Gene_6220 | 91 | 98.5 | BAC79030.1 | NDP-glucose-4,6-dehydratase |
| Gene_6221 | 80 | 99.4 | BAC79031.1 | NDP-deoxyglucose-2,3-dehydratase |
| Gene_6222 | 89 | 100.0 | BAC79032.1 | N-methyl transferase |
| Gene_6223 | 81 | 91.2 | BAC79033.1 | NDP-4-keto-6-deoxyhexose reductase |
| Gene_6224 | 85 | 98.8 | BAC79035.1 | NADH:FMN oxidoreductase |
| Gene_6225 | 87 | 73.0 | BAC79036.1 | stereospecific keto reductase |
| Gene_6226 | 91 | 99.6 | BAC79036.1 | stereospecific keto reductase |
| Gene_6227 | 91 | 100.0 | BAC79037.1 | pathway-specific transcriptional activator (SARP) |
| Gene_6228 | 89 | 100.0 | BAC79038.1 | unknown (ActVI-A homologue) |
| Gene_6229 | 73 | 78.7 | BAC79039.1 | enoyl reductase |
| Gene_6230 | 88 | 100.0 | BAC79039.1 | enoyl reductase |
| Gene_6231 | 87 | 99.7 | BAC79040.1 | C-glycosyl transferase |
| Gene_6232 | 83 | 99.5 | BAC79041.1 | pyran ring cyclase/possible exporter (ActVI-3 homologue) |
| Gene_6233 | 95 | 100.0 | BAC79042.1 | keto reductase (KR) |
| Gene_6234 | 84 | 100.0 | BAC79043.1 | oxygenase/hydroxylase |
| Gene_6235 | 93 | 100.0 | BAC79044.1 | ketosynthase (KS) alpha subunit |
| Gene_6236 | 89 | 99.8 | BAC79045.1 | ketosynthase (KS) beta subunit |
| Gene_6237 | 86 | 99.3 | BAC79046.1 | second ring cyclase (CYC) |
